# The Effectiveness of In-house Clear Aligners and Traditional Fixed Appliances in Achieving Good Occlusion in Complex Orthodontic Cases: A Randomized Control Clinical Trial

**DOI:** 10.7759/cureus.30147

**Published:** 2022-10-10

**Authors:** Samer T Jaber, Mohammad Y Hajeer, Ahmad S Burhan

**Affiliations:** 1 Department of Orthodontics, Al-Wataniya Private University, Hama, SYR; 2 Department of Orthodontics, University of Damascus Faculty of Dentistry, Damascus, SYR

**Keywords:** study models, treatment duration, effectiveness, orthodontic, premolars extraction, complex cases, crowding, class i, in-house clear aligners, clear aligners

## Abstract

Objective: To compare the effectiveness and efficiency of the in-house clear aligners with the traditional fixed appliances in treating premolar-extraction-based complex cases.

Materials and methods: A single-centered, 2-arm parallel-group randomized controlled clinical trial was conducted on thirty-six (12 males, 24 females; mean age: 21.24 ± 2.33) patients with severe crowding who required orthodontic treatment with four first premolars extraction. The patients were equally and randomly divided into two groups: The in-house clear aligners (CA) group and the fixed appliances (FA) group. All the measurements were made on the pre-and post-treatment dental cast models. The effectiveness was evaluated using Little's irregularity index (LII) and the Peer Assessment Rating index (PAR). The efficiency was evaluated by studying the treatment duration. Two sample t-tests and chi-square tests were used to test for significant differences between the two groups. Bonferroni correction was applied, and the adjusted alpha level was set at 0.006.

Results: Before treatment, there were no significant differences between the two groups regarding LII in the upper and lower jaws (p˃0.006). After treatment, the mean LII decreased in both groups, with no significant differences between the two studied groups (p˃0.006). There were no significant differences in all studied PAR domains between the two groups (p˃0.006). The mean score reduction in the CA group was 28.39 (±8.51) points, whereas it was 26.39 (±5.76) points in the FA group, with no significant differences between the two groups. All the patients in this study were improved. However, a great improvement was achieved in 88.9% of the patients in the CA group and 91.7% in the FA group, with no significant differences between them (p=0.674). The average treatment duration in the CA group was 23.27 (±5.28) months, whereas the average was 26.20 (±5.27) in the FA group, with no significant difference between the two groups.

Conclusion: In-house clear aligners can be effective as fixed appliances in achieving good occlusion when treating complex orthodontic cases when suitable teeth movement protocol is used.

## Introduction

With the increased demand for orthodontic treatment among adult patients, clear aligners (CA) have experienced a shift in the market [1]. The most significant benefit of this technique is its improved aesthetics and comfort for the patients compared to traditional fixed appliances (FA) [2]. The concept of moving teeth using transparent thermoplastic appliances was introduced in the early 1940s by Kesling [3]. With the development of computer-aided design (CAD)/computer-aided manufacturing (CAM) technologies and the Invisalign system by Align Technology, Inc. in the late 1990s, CA has witnessed a dramatic development until today [4,5]. Lately, the availability of orthodontic treatment planning software alongside three-dimensional (3D) scanners and a wide range of validated 3D validated printers and printing materials in dental clinics has encouraged clinicians to manufacture their patients' aligners in-house, in other words, in their clinics without the need to communicate with CA providers [6,7].

Several occlusal indices have been developed to quantitively assess orthodontic treatment outcomes to help assess the effectiveness of the used technique [8]. The American Board of Orthodontics Objective Grading System (ABO-OGS) and the Peer Assessment Rating (PAR) index may be among the most popular assessment tools in orthodontics [9,10]. The ABO-OGS evaluates the treatment outcomes using the post-treatment dental casts and panoramic radiographs [11], whereas the PAR index uses the pre- and post-treatment dental casts, and differences in the PAR scores reflect the improvement achieved in the treated cases, which is an advantage not found when using the ABO-OGS [12].

The efficiency and effectiveness of clear aligners have been studied copiously in mild and moderate malocclusion cases, whereas little attention has been paid to assessing clear aligners in more complex malocclusion cases. Sachdev et al. evaluated the accuracy of tooth movement with in-house clear aligners in treating mild non-extraction cases using computerized superimposition between predicted and achieved digital models. The study reiterated that in-house clear aligners benefit non-complex cases, and the treatment goals can be achieved at a lower cost [13]. Li et al. [9] and Gaffuri et al. [14] found that clear aligners provided by Invisalign® were effective in treating complex cases requiring the extraction of four first premolars after assessing the cases using ABO-OGS. A recent umbrella review [15] found that CA was effective for treating mild to moderate malocclusions with a shorter treatment time, while it was associated with inferior outcomes when treating complex cases compared to FA. The review concluded that more high-quality randomized controlled trials (RCTs) support the evidence.

Therefore, this trial aimed to compare the effectiveness and efficiency of the in-house clear aligners with the traditional fixed appliances in treating premolar-extraction-based complex cases by comparing Littles's irregularity index, PAR index, and treatment duration. The null hypothesis was that there were no statistically and clinically significant differences between the in-house clear aligners and the fixed appliances regarding the LII, studied PAR scores, and the treatment duration of the treated patients.

## Materials and methods

Trial design, registration, and ethical approval

This study was a single-center, 2-arm parallel-group RCT. It was conducted at the Department of Orthodontics, University of Damascus Dental School, Damascus, Syria. The protocol of this study was reviewed and approved by the Local Research Ethics Committee of the University of Damascus Dental School (Approval no. UDDS-5864-2019PG/SRC3901) and was funded by the University of Damascus Dental School Post-graduate Research Budget (Ref no. 841973194DEN). The study was registered at the in ClinicalTrials.gov database (Identifier: NCT05500456). No changes in the study protocol have occurred after trial commencement. This study was reported according to the guidelines of the CONSORT statement [16].

Sample size calculation

The sample size of this study was calculated using Minitab™ (version 21; Minitab, LLC, State College, Pa, USA), and the intended statistical test was a ''two-sample t-test''. The following assumptions were used: the smallest difference requiring detection in the weighted post-PAR score was 3 points, a standard deviation of 2.23 taken from a previous study [17], and the alpha level and study power were set at 0.05 and 0.95, respectively. The analysis revealed that 32 patients (16 in each group) were required. Four patients were added in case of dropouts. Finally, 36 patients (18 in each group) were recruited.

Patient recruitment and entry into the trial

An evaluation of 101 patients who had been referred to the Department of Orthodontics, University of Damascus Dental School, Damascus, Syria, with a primary diagnosis of severe dental crowding. Thirty-six patients were randomly and equally allocated into two groups: the Clear aligners group (CA) and the conventional fixed appliances group (FA). Information sheets were disturbed to all participants and written informed consent forms were collected. Inclusion criteria were: (1) Age ranging from 18 to 25 years, (2) Class I skeletal and dental malocclusion, and (3) severe crowding (more than 6 mm of tooth size-arch length discrepancy), which require orthodontic treatment with upper and lower first premolars, (4) No congenitally missing or extracted teeth (except for the third molars), (5) No history of trauma to the maxillofacial region or surgical interventions. Exclusion criteria were: (1) Previous orthodontic treatment, (2) Patients with psychological abnormalities, (3) Patients with systematic diseases, and (4) Patients with known allergies to latex and plastic.

Randomization, allocation concealment, and blinding

Randomization and sequence generation was done by one of the academic staff not involved in this study. With an allocation ratio of 1:1, a computer-generated list of random numbers was exported by Minitab® (version 17, Minitab, LLC, State College, PA). The allocation sequence was concealed using opaque, sealed, sequentially numbered envelopes opened only before the onset of the treatment. Blinding of the investigator and participants were not possible because of the nature of the intervention; therefore, blinding was only applicated to the outcome assessment and statistical analysis.

Interventions: clear aligners (CA) group

In both groups, the orthodontic treatment for the patients was provided by the same principal investigator (STJ) under the supervision of one of the co-authors of this manuscript (MYH). The first step was preparing digital working models for each patient enrolled in this group by taking additional silicon impressions (Zhermack, elite HD+, Rome, Italy) for the patients, pouring them with gypsum, and scanning the models with a desktop 3d dental scanner (Identica Hybrid; MEDIT, Seoul, Korea). Zero aligners were thermoformed on a 3D printed replica of the digital working models. These aligners aim to allow patients to get used to this treatment technique, prevent minor teeth movements after premolars extraction, and eliminate residual adaptations due to scanning and printing procedures [7]. Scanned files were imported to the 3Shape Orthoanalyzer™ 2019 software (3Shape, Copenhagen, Denmark) for virtual treatment planning. Models were prepared in a horse-shoe shape, then segmentation of the teeth was done after superimposition of the models and the cone-beam computed tomography (CBCT) for precise determination of the longitudinal axis and the center of resistance of each tooth. Virtual setups for the cases were prepared using a unique teeth movement protocol developed by the principal investigator (STJ) and the main supervisor (MYH) for the virtual setup of severe crowding cases.

A protocol developed by the research team titled 'stepwise activated movements by multiple enhanced re-anchorage (SAMMER)' was used in the treatment planning. This protocol divided teeth movements into three stages. In the first stage (decrowding), leveling and alignment movements of the crowded anterior teeth were done using the posterior teeth (2nd premolars and molars) as an anchorage unit. In the first stage, canines were moved first, followed by the lateral incisors, and finally, the central incisors. Dentoalveolar protrusion of the incisors must be corrected in this stage if needed. In the second stage (space closure), the class I canine and molar relationship was achieved by allowing second premolars and molars movement if needed. In the last stage (fine-tuning), the final movements for overcorrection, closure of any residual spaces, and taking the final alignment of the teeth with the use of posterior intercuspation techniques if needed. Attachments were added to the teeth where needed. The tooth movement maximum threshold per aligner was set at 0.25 mm for the transitional movements and 2° for the rotational movements [13]. According to this protocol and threshold, each virtual treatment plan was subdivided into sub-setups which were exported for 3D printing to the validated printer (i3 MK3; Prusa, Czech), and clear aligners were formed for each model using the 0.762 mm Taguls™ premium aligner sheets (Taguls, Vedia solutions, Mumbai, India) and the Biostar® thermoforming device (Biostar, Scheu-dental, Iserlohn, Germany). Aligner trimming, polishing, disinfecting, packing, and delivering to the patient were done for each case in this group. In the first appointment, the first aligners were applied after attachment bonding using the attachments template; instructions were given to wear the aligners 20 hours a day, seven days a week. Each aligner was changed every two weeks, and each patient was seen for a follow-up every four weeks to check for aligner fit, attachment stability, and cooperation. Additional refinement aligners were provided if treatment outcomes were not accomplished.

Conventional fixed appliances (FA) group

Conventional orthodontic treatment with vestibular fixed appliances was applied to the patients in this group. Application of the appliances had been made one week after the premolars extraction. Brackets with a 0.22-inch slot height and MBT prescription (Master Series®, American Orthodontics™, Sheboygan, WI) were used with a transpalatal arch (TPA) and Nance button in the upper jaw and lingual arch in the lower jaw as anchorage devices. The following sequence of archwires was used during the leveling and alignment phase: 0.014-inch Nickle-Titanium (NiTi), 0.016-inch NiTi, 0.016*0.022-inch NiTi, 0.017*0.025-inch NiTi, and then 0.019*0.025-inch stainless steel wire [18]. When the crowding started to decrease, the next wire was applied, considering that it would not apply excessive force on the teeth, and the previous wire was passively inserted into the slots of all brackets. The patients were seen every four weeks for follow-up. 

Definition of the endpoint of treatment

The treatment in the FA group was considered complete when patients had undergone all different stages of orthodontic treatment, including detailing and stabilization. In the CA group, the treatment was considered complete when all the prescribed aligners were fully used in addition to achieving satisfaction with the final occlusion and dental relationships by two of the three main persons involved in this treatment: (1) the patient per se, (2) the principal researcher who treated the case, and/or (2) the main supervisor of this trial.

Outcome measures

All the measurements included in this study were made by a calibrated examiner (the third author), who was blinded to the treatment group he was scoring for. All the measurements were made on the pre-and post-treatment dental cast models. 

Little's Irregularity Index (LII)

LII assesses the crowding degree of the anterior teeth by measuring the linear displacements in the horizontal plane between the contact points of the anterior teeth from the mesial surface of one canine to the contralateral one. The LII score is the sum of these linear measurements, and a higher score value indicates more severe irregularities of the anterior teeth [19].

The PAR Index

The weighted PAR index was used in this study, which assesses the following components: maxillary and mandibular anterior segment alignment, anterior-posterior, transverse, and vertical posterior teeth occlusion, overjet, overbite, and the midline [20]. Measurements were made using the PAR index ruler. The score of each component is summed up to a total PAR score. A higher PAR score implicates a greater case complexity, and differences in the results before and after the orthodontic treatment represent the improvement degree. A reduction in the PAR score of at least 30% classifies the case as 'improved'. Reducing 22 points or more in the weighted PAR score classifies the case as 'greatly improved'. Improvements smaller than 30% are declared 'worse or no different' [21].

Treatment Duration

The efficiency of the two techniques was evaluated by comparing the treatment duration, which was recorded by months in both groups. It was calculated from the first day the active orthodontic treatment started (first aligner in the CA group, first wire in the FA group). 

Statistical analysis

Statistical analysis was performed using the SPSS (version 25.0; IBM, Armonk, NY, USA). Chi-square tests were used to determine whether there were any differences in the distributions between the two groups regarding gender, whereas 2 sample t-tests were used for age and LII. Anderson-Darling Normality tests were used to test the normality of distributions. Two sample t-tests were used to test for significant differences between the two groups regarding LII treatment duration and PAR index components, except for the comparison for cases improvement where chi-square tests were used. Because of multiple comparisons, Bonferroni correction was applied when the comparisons of the LII and PAR index were made between the studied groups, and the adjusted alpha level was set at 0.006. 

The error in the method

Twenty models were randomly chosen, and the LII and the PAR index were rescored after a four-week interval. For the intraexaminer reliability (random error) determination, the intraclass correlation coefficients (ICCs) were used, whereas the paired t-test was used to identify any systematic error.

## Results

Participant recruitment and entry into this trial

**Figure 1:**
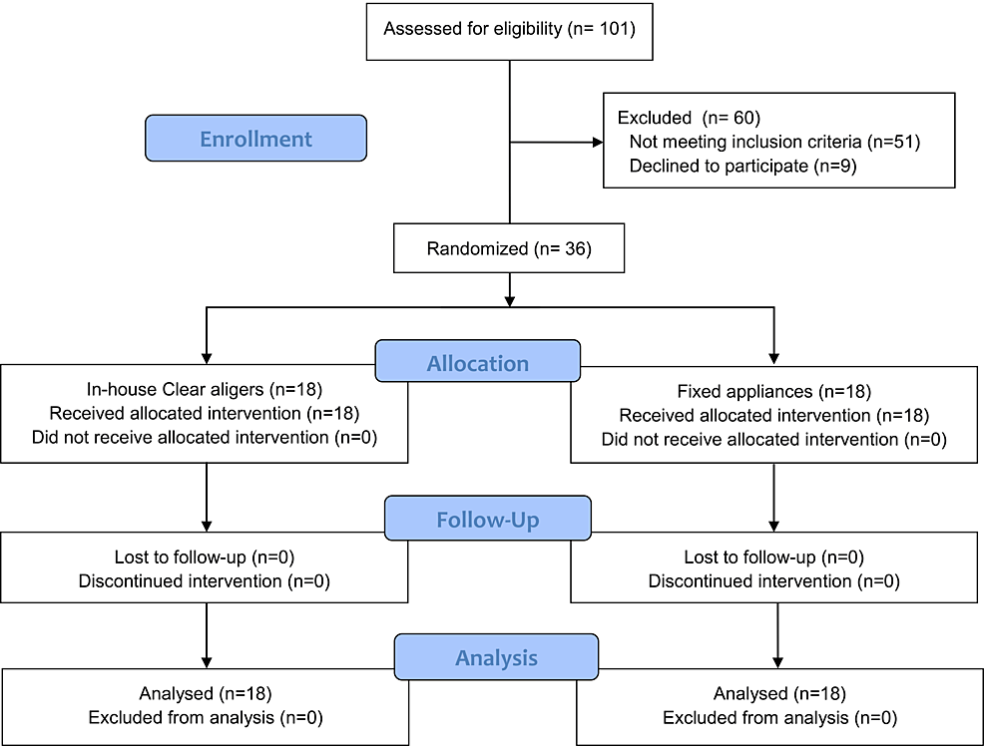
Consolidated Standards of Reporting Trials (CONSORT) flow diagram of patients' recruitment and follow-up.

Recruitment of the participants started in June 2019 and ended in January 2020. The CONSORT participants' flow diagram is shown in Figure 1. 101 patients were assessed for eligibility, and 60 patients were excluded for not meeting the inclusion criteria or declining to participate in the study. Thirty-six (12 males and 24 females) of the 41 candidate patients were randomly selected. Then, they were randomly distributed into two groups: 18 patients (5 males, 13 females) with an age range of 21.30 (±2.29) years in the CA group and 18 patients (12 males, 24 females) with an age range of 21.18 (±2.44) years in the FA group. No patient was lost to follow-up in both groups. The baseline characteristics before starting the treatment are given in Table 1. 

**Table 1:** Basic sample characteristics (age, gender, and Little's index) CA: Clear Aligners group, FA: Fixed Appliance group, † Employing chi-square test, ‡ Employing two-sample t-test. *Significant difference between groups (p˂0.05).

Variable		CA group (n=18)	FA group (n=18)	Both groups n=36	P-value
Gender † Number (%)	Male	5 (27.8%)	7(38.9%)	12 (33.3%)	0.480
	Female	13 (72.3%)	11 (61.2%)	24 (66.7%)	
Age in years ‡ (Mean ± Standard deviation)		21.30 ± 2.29	21.18 ± 2.44	21.24 ± 2.33	0.877
Little's index ‡ Mean ± Standard deviation	Upper	12.94 ± 2.14	11.19 ± 2.89	12.06 ± 2.66	0.068
	Lower	10.55 ± 3.49	11.66 ± 2.72	11.11 ± 3.14	0.780

The error in the method

The ICCs ranged from 0.951 to 0.995, which indicated high interexaminer reliability for the measurements. Paired t-test showed that there were no significant differences between the two measurements done (p˃0.05), indicating that the systematic errors were small and insignificant.

Main results of outcome assessment

Little's Irregularity Index (LII)

Before starting the treatment, the mean LII in the upper jaw was 12.94 (±2.14) and 11.19 (±2.89) in the CA and FA groups, respectively. In the lower jaw, the mean LII was 10.55 (±3.49) in the CA group and 11.66 (±2.72) in the FA group. There were no significant differences between the two groups regarding LII in the upper and lower jaws (Table 1). After treatment, the mean LII decreased in both groups. For the CA group, the mean LII was 0.58 (±0.69) and 0.69 (±0.87) for the upper and lower jaws, respectively. In the FA group, the mean LII for the upper jaw was 0.47 (±0.36), whereas it was 0.61 (±0.47) in the lower jaw. There were no significant differences in the LII for the upper and lower jaws between the two studied groups (Table 2).

**Table 2:** Descriptive statistics of the LII and PAR index and the result of the significant tests CA: Clear Aligners group, FA: Fixed Appliance group, LII: Little's irregularity index, UANT: Upper anterior segment alignment, LANT: Lower anterior segment alignment, AP: Anterior-posterior occlusion, TRANS: Transverse occlusion, VERT: Vertical occlusion, OJ: Overjet, OB: Overbite, MID: Midline, WPAR: Weighted PAR, RED: Reduction percent. † Employing a two-sample t-test, ‡ Employing a chi-square test. *Bonferroni's correction was used to adjust the significance level to 0.006.

Variable		CA group (n=18)		FA group (n=18)		CA vs. FA		
		T0	T1	T0	T1	T1		
		Mean ± Standard deviation	Mean ± Standard deviation	Mean ± Standard deviation	Mean ± Standard deviation	Mean difference	95% CI	p-value
LII	Upper Jaw†	12.94 ± 2.14	0.58 ± 0.69	11.19 ± 2.89	0.47 ± 0.36	0.11	(-0.268; 0.490)	0.551
	Lower Jaw†	10.55 ± 3.49	0.69 ± 0.87	11.66 ± 2.72	0.61 ± 0.47	0.08	(-0.399; 0.566)	0.725
PAR index	UANT†	7.27 ± 1.87	0.44 ± 1.87	6.55 ± 2.12	0.05 ± 0.23	0.38	(0.114; 0.663)	0.008
	LANT†	6.22 ± 1.39	0.83 ± 0.98	6.27 ± 2.13	0.22 ± 0.54	0.61	(0.065; 1.157)	0.030
	AP†	1.05 ± 1.16	1.16 ± 0.61	2.16 ± 1.09	1.22 ± 0.87	-0.05	(-0.573; 0.461)	0.828
	TRANS†	0.55 ± 0.92	0.05 ± 0.23	0.66 ± 0.84	0.33 ± 0.76	-0.27	(-0.672; 0.117)	0.157
	VERT†	1.22 ± 0.94	0.27 ± 0.66	0.72 ± 0.82	0.38 ± 0.60	-0.11	(-0.545; 0.322)	0.605
	OJ†	13.00 ± 6.90	0.66 ± 1.94	9.00 ± 5.54	0.66 ± 0.84	0.00	(-1.314; 1.314)	1.00
	OB†	1.11 ± 1.84	0.66 ± 1.18	0.66 ± 1.18	0.33 ± 1.02	0.33	(-0.420; 1.087)	0.375
	MID†	3.55 ± 2.33	1.33 ± 1.94	4.44 ± 2.70	1.33 ± 2.37	0.00	(-1.473; 1.473)	1.00
	WPAR†	33.89 ± 8.01	5.50 ± 2.85	30.44 ± 6.69	4.05 ± 2.96	1.44	(-0.527; 3.416)	0.146
	Score reduction†	28.39 ± 8.51		26.39 ± 5.76		2.00	(-2.950; 6.950)	0.416
	RED%†	87.08 ± 8.37		83.52 ± 9.15		-3.56	(-9.510; 2.380)	0.232
	Improved cases‡ Number (%)	18/18 (100%)		18/18 (100%)		-	-	1.00
	Great Improvement‡ Number (%)	14/18 (88.9%)		15/18 (91.7%)		-	-	0.674

The PAR Index

An improvement was observed in all eight components of the PAR score in both groups (as shown in the previous table). The most prominent improvement in both groups was seen in the weighted overjet component, whereas the slightest improvement was in the vertical occlusion component. There were no significant differences in all studied PAR domains between the two groups (p˃0.006). In the CA group, the mean score reduction was 28.39 (±8.51) points, whereas it was 26.39 (±5.76) points in the FA group, with no significant differences between the two groups. In both groups, all the patients were improved; i.e., at least a 30% reduction in the weighted PAR score was achieved after treatment. However, a great improvement was achieved in 88.9% of the patients in the CA group and 91.7% in the FA group, with no significant differences between them (p=0.674).

Treatment Duration

The average treatment duration in the CA group was 23.27 (±5.28) months, whereas the average was 26.20 (±5.27) in the FA group, with no significant difference between the two groups (p=0.123; Table 3).

**Table 3:** Descriptive statistics of the treatment duration and the result of the significant test CA: Clear Aligners group, FA: Fixed Appliance group. † Employing a two-sample t-test. *Significant difference between groups (p˂0.05)

Variable	CA group (n=18)		FA group (n=18)		CA vs. FA		
	Mean ± Standard deviation	SE Mean	Mean ± Standard deviation	SE Mean	Mean difference	95% CI	p-value
Treatment Duration†	23.27 ± 5.82	1.37	26.20 ± 5.27	1.24	-2.93	(-6.70; 0.83)	0.123

Harms

No significant harm or untoward effects were observed in both groups during this study.

## Discussion

To date, only two previously published RCTs have evaluated the CA's effectiveness in the treatment of complex cases, including premolars extraction, and both of them used aligners provided by Invisalign®. The current RCT is the first one to assess the effectiveness and efficacy of in-house clear aligners in treating severe crowding cases as one of the complex orthodontic cases requiring the extraction of the first premolars. The distribution of the patients in the study groups was well-balanced according to age, gender, and LII. The age range of the patients was limited between 18-25 years as the largest percentage of adult orthodontic patients are young adults aged 18-25 years [22].

All the individual components of the PAR index were reduced after treatment except for the anterior-posterior component in the CA group, which was increased by less than 1 point after treatment. There were no differences between the eight components of the PAR index after treatment between the two groups, and the mean points differences were always lesser than a single point.

The average weighted post-treatment PAR score of the CA group was higher than that of the FA group by 1.44 points, with no significant difference. The PAR score was reduced by more than 25 points in both groups after treatment, and the reduction was 2 points higher in the CA group than that of the FA group; this also was not statistically different.

Considering the percentage of improvement of the weighted PAR score, all the patients in both groups were classified as ''Improved'' after treatment, whereas a ''Great improvement'' was noticed in more than 88% of the patients in both groups (88.9% in CA, 91.7% in FA) with no significant differences between them. These results suggested that the in-house aligners used in this study could achieve a comparable good result in extraction cases as the conventional fixed appliances, and this comes in line with the findings of Li et al. [9] and Gaffuri et al. [14] who found that Invisalign and fixed appliances can effectively treat complex cases requiring the extraction of first premolars.

This study also evaluated the efficiency of in-house clear aligners by considering treatment duration. There were no significant differences between the treatment duration of the studied groups. In the CA group, the duration was, on average, 2.9 months faster than in the FA group. This result differs from the previous studies [9,14], which found that the Invisalign treatment was 44% longer than the fixed appliances treatment in four premolars extraction cases. This could be attributed to the difference between the treatment planning protocol used in this study and the protocols used by Invisalign for extraction cases, like the G6 Align protocol. The SAMMER protocol reveals the crowding by moving the front teeth gradually. The G6 protocol retracts the canines in the first step to the third of the extraction space without applying movements on the crowded front teeth. In the next step, retraction of the front teeth starts with the canines continuing their retraction movement [23]. This increases the number of aligners required with Align's protocol compared to the protocol used in this study, leading to more extended treatment duration.

Limitations

The current study employed the PAR index to compare the occlusal outcomes between the FA and CA appliances. Another insight into the achieved changes could have been evaluated using other orthodontic indices, such as the American Board of Orthodontics Objective Grading System (ABO-OGS), which includes other components not analyzed with the PAR index. The study included patients with severe crowding who were treated with four-premolar extractions. It might be beneficial to study other extraction-based treatment plans for patients with bimaxillary protrusion, Class II, and Class III malocclusions. The treatment outcomes were compared between the two groups directly following appliance removal. Therefore, long-term stability should be assessed. Additionally, attention should be paid to comparing in-house aligners with those produced by well-known commercial companies regarding treatment efficiency and effectiveness.

## Conclusions

There were no significant differences between the CA and FA groups for any of the components of the PAR index after the end of treatment. The in-house clear aligners can effectively treat complex orthodontic cases, including premolar-extraction-based treatments if a suitable teeth movement protocol is used. There was no difference in the treatment duration with in-house clear aligners and fixed appliances. 
